# Influence of Baseline Itch Severity on Treatment Outcomes With Difelikefalin in Adults With Moderate‐to‐Severe Pruritus Receiving Maintenance Haemodialysis: An Exploratory Analysis

**DOI:** 10.1111/jorc.70017

**Published:** 2025-05-06

**Authors:** Kieran McCafferty, Thilo Schaufler, Pablo Molina, Murray Lowe, Warren Wen, Daniel E. Weiner

**Affiliations:** ^1^ Barts Health NHS Trust London UK; ^2^ CSL Vifor Glattbrugg Switzerland; ^3^ Department of Nephrology FISABIO, Hospital Universitari Dr Peset Universitat de València València Spain; ^4^ Cara Therapeutics Stamford Connecticut USA; ^5^ William B. Schwartz, MD, Division of Nephrology Tufts Medical Center Boston Massachusetts USA

**Keywords:** difelikefalin, haemodialysis, pruritus, quality of life, tolerability

## Abstract

**Background:**

Difelikefalin is well‐tolerated and reduces itch among adults undergoing haemodialysis (HD), with chronic kidney disease‐associated pruritus (CKD‐aP).

**Objective:**

This study aims to explore the influence of baseline itch severity on difelikefalin treatment outcomes.

**Design:**

Study 3105 (NCT03998163) was a 12‐week, phase 3, single‐arm, open‐label trial assessing safety and effectiveness of difelikefalin 0.5 µg/kg. We report key endpoints from 3105 by baseline itch severity, determined using the Worst Itching Intensity Numerical Rating Scale (WI‐NRS [moderate: WI‐NRS < 7; severe: WI‐NRS ≥ 7]).

**Participants:**

Adult participants undergoing maintenance HD (*n* = 222) with mild‐to‐moderate CKD‐aP (WI‐NRS score ≥ 5 at baseline).

**Measurements:**

The primary endpoint of 3105 was safety; secondary endpoints included reduction in itch intensity (WI‐NRS), and improvements in itch‐related quality of life (QoL; 5‐D itch scale) and sleep quality (Sleep Quality Numerical Rating Scale).

**Findings:**

Mean (SD) age was 57.1 (13.3) years; mean (SD) baseline WI‐NRS scores were 6.0 (0.5) and 8.3 (0.9) for participants with moderate (*n* = 70/222) or severe (*n* = 152/222) itch, respectively. No treatment‐related deaths occurred, and there were no safety concerns according to baseline itch severity. By week 12, both groups reported residual ‘mild itch’ according to mean (SD) WI‐NRS scores (moderate: 2.9 [2.2]; severe: 3.1 [2.3]). Approximately one in four participants demonstrated ‘complete response’ in itch reduction (moderate: 27.1%; severe: 25.0%). Clinically relevant improvements in itch‐related QoL and sleep quality occurred among both subgroups.

**Conclusions:**

Difelikefalin was well‐tolerated and effective in reducing itch in participants with moderate and severe baseline itch, supporting its broad use in a range of individuals on HD with CKD‐aP.

## 1 Introduction

Up to 80% of individuals receiving maintenance haemodialysis (HD) for chronic kidney disease (CKD) experience CKD‐associated pruritus (CKD‐aP), with symptoms reaching ‘moderate‐to‐severe’ in approximately 40% (Kim and Pollock 2021). The clinical presentation of CKD‐aP is highly heterogeneous in terms of severity, onset around HD sessions and distribution of itch (Kim and Pollock 2021). Individuals who are at least ‘moderately bothered’ by CKD‐aP experience reduced sleep quality, impaired health‐related quality of life (QoL), depression and poorer clinical outcomes, compared to those experiencing mild CKD‐aP (‘somewhat bothered’ by itch) (Rayner et al. 2017; Rehman et al. 2019; Sukul et al. 2021). Furthermore, observational evidence from the Dialysis Outcomes and Practice Patterns Study demonstrated that the likelihood of withdrawal from HD, or a missed HD session, was higher among those with increased CKD‐aP severity (Sukul et al. 2021). Thus, there is a clinical need to provide viable treatment options to relieve the physical and mental burden of CKD‐aP and to improve outcomes for those living with this condition.

Although difelikefalin is approved for use in adults with moderate‐to‐severe CKD‐aP undergoing maintenance HD, the influence of baseline itch severity on difelikefalin treatment outcomes is not yet fully elucidated. Understanding the potential implications of differences in baseline itch severity between those with ‘moderate’ versus ‘severe’ CKD‐aP could provide valuable insights to aid healthcare providers with informed decision‐making when treating individuals with different severities of CKD‐aP. Therefore, the aim of this exploratory analysis from Study 3105 was to assess the safety and effectiveness of difelikefalin among adults on maintenance HD, classed as having moderate‐to‐severe CKD‐aP, according to baseline itch severity.

## 2 Literature Review

As mentioned earlier, difelikefalin is a potent and highly selective kappa‐opioid receptor agonist, which is approved for use as a first‐in‐class treatment for moderate‐to‐severe CKD‐aP in adults undergoing HD (European Medicines Agency 2022; US Food and Drug Administration 2021). The randomised, phase 3, placebo‐controlled KALM‐1 (NCT03422653) and KALM‐2 (NCT03636269) trials demonstrated that difelikefalin significantly reduced itch intensity, as assessed by the Worst Itching Intensity Numerical Rating Scale (WI‐NRS) and improved itch‐related QoL compared to placebo, with an acceptable safety profile (Fishbane et al. 2020; Topf et al. 2022). Safety findings were further validated among individuals with CKD‐aP undergoing HD in the phase 3, single‐arm, open‐label trial, Study 3105 (NCT03998163) (Weiner et al. 2022). Furthermore, three out of four participants in 3105 reported a clinically meaningful reduction in itch intensity following 12 weeks of treatment with difelikefalin (Weiner et al. 2022). Clinically relevant improvements in itch‐related QoL were also reported in 70% and 63% of participants, according to the 5‐D itch and Skindex‐10 scales, respectively; and 66% reported clinically relevant improvements in sleep quality, as per the Sleep Quality Numerical Rating Scale (SQ‐NRS) (Weiner et al. 2022).

## 3 Materials and Methods

### 3.1 Study Design

Full methodology from Study 3105 (NCT03998163) has previously been published (Weiner et al. 2022). Briefly, Study 3105 was a global, phase 3, multicentre, open label, single‐arm intervention trial assessing the safety and effectiveness of difelikefalin in adults on maintenance HD with moderate‐to‐severe CKD‐aP at baseline. Participants received difelikefalin 0.5 µg/kg as an intravenous bolus after each HD session (~3 times weekly) over a period of up to 12 weeks (Supporting Information S1: Figure S1). Study 3105 was conducted across 43 centres in the United States (*n* = 31) and Eastern Europe (*n* = 12) between May 2019 and March 2020.

Baseline itch severity was determined using the weekly mean WI‐NRS score during the run‐in period. The WI‐NRS is a widely used, validated tool for quantifying itch intensity (Kumagai et al. 2010; Mathur et al. 2010; Ständer et al. 2013). Scores range from 0 to 10, with higher scores indicating greater itch severity. Changes in this score correlate with several clinically meaningful itch‐related QoL measures, making it a reliable metric in pruritus studies (Fishbane et al. 2020; Vernon et al. 2021). This rating scale was additionally selected for its simplicity and ease of use within the constraints of a busy clinical setting.

In this exploratory subgroup analysis, key endpoints from Study 3105 were analysed according to baseline itch severity (moderate CKD‐aP subgroup: WI‐NRS < 7; severe CKD‐aP subgroup: WI‐NRS ≥ 7).

### 3.2 Study Population

Full eligibility criteria for Study 3105 are listed in Supporting Information S2: Table S1. Eligible participants were between 18 and 85 years old with a mean WI‐NRS score of ≥ 5 at baseline, indicating moderate or severe CKD‐aP, and receiving 3 times weekly in‐centre maintenance HD for at least 3 months before screening. Participants were not eligible if they were scheduled to receive a kidney transplant during the study period, suffered from pruritus not attributable to CKD, or had any concomitant disease that may have interfered with the study or caused undue risk.

### 3.3 Endpoints

Key safety and effectiveness endpoints from Study 3105 reported in this analysis are listed in Table 1. As well as being used to define itch severity at baseline, improvements in itch intensity by week 12 were measured using the WI‐NRS. The 5‐D itch scale total score was used to determine itch‐related QoL, an element of overall health‐related QoL (Elman et al. 2010). Scores range from 5 to 25, with higher scores indicating worsening itch intensity and poorer itch‐related QoL. Sleep quality was assessed using the SQ‐NRS and 5‐D itch sleep disability question (5D SDQ) to determine how much a patient's itch had interfered with their sleep in the preceding 24 h (SQ‐NRS: 0, did not interfere to 10, completely interfered; 5D SDQ: 1, never affects sleep to 5, delays falling asleep and frequently wakes me up at night). Similar to the WI‐NRS, SQ‐NRS and 5‐D itch scores were chosen as they have previously been successfully used in clinical studies exploring the treatment effects of difelikefalin on CKD‐aP as a means of measuring improvements in itch intensity and itch‐related QoL (Weiner et al. 2022).

**Table 1:** Summary of safety and effectiveness endpoints reported in this analysis.

	Outcome	Endpoints assessed by baseline itch severity
**Primary objective**		
Safety	All adverse events	Number of TEAEs; percentage of participants experiencing a TEAE Number of serious TEAEs; percentage of participants experiencing serious TEAE Number of AESI; percentage of participants experiencing an AESI Number (%) of participants experiencing AESI by category
**Secondary objectives**		
Reduction in itch intensity	WI‐NRS	Percentage of participants achieving a ≥ 3 or ≥ 4‐point improvement from baseline in the weekly mean of the daily 24‐h WI‐NRS at Week 12 Percentage of complete responders (≥ 75% of weekly mean WI‐NRS scores equal to 0 or 1) Change from baseline in the weekly mean of the 24‐h WI‐NRS score to week 12; mean score at week 12
Improvements in itch‐related QoL	5‐D itch	Percentage of participants achieving a ≥ 5‐point improvement from baseline in 5‐D itch scores at week 12 Change from baseline in the mean 5‐D itch score to week 12; mean score at week 12
Improvements in itch‐related sleep quality	SQ‐NRS 5D SDQ	Percentage of participants achieving a ≥ 3 or ≥ 4‐point improvement from baseline to week 12 in the weekly mean of the 24‐h SQ‐NRS score Percentage of complete responders (all SQ‐NRS scores equal to 0) at week 12 Change from baseline to week 12 in the weekly mean of the 24‐h SQ‐NRS score; mean score at week 12 Percentage of participants achieving a ≥ 1‐point improvement from baseline to week 12 in the mean 5D SDQ score Change from baseline to week 12 in the mean 5D SDQ; mean score at week 12

*Note:* WI‐NRS scale (1–10), higher score indicating greater itch intensity; 5‐D itch scale total score (5–25), higher scores indicate worsening itch intensity and itch‐related QoL; SQ‐NRS (scores range from 0 [did not interfere with sleep] to 10 [completely interfered with sleep]); 5D SDQ (scores ranging from 1 [never affects sleep] to 5 [delays falling asleep and frequently wakes me up at night]).

Abbreviations: 5D SDQ, 5‐D itch sleep disability question; AESI, adverse event of special interest; QoL, quality of life; SQ‐NRS, sleep quality numerical rating scale; TEAE, treatment emergent adverse event; WI‐NRS, worst itching intensity numerical rating scale.

Safety was evaluated through continual reporting of any treatment emergent adverse events (TEAEs), including AEs of special interest (AESI), captured from the time an individual signed the consent form until the Follow‐up Visit (7–10 days after End of Treatment/Early Termination Visit). Safety data are presented for the moderate CKD‐aP subgroup, the severe CKD‐aP subgroup, and the overall study population from Study 3105 (Weiner et al. 2022).

Regarding efficacy, the thresholds for deeming improvements (score reductions) in each of the patient‐reported outcomes (PROs) described here as clinically relevant were based on previously published findings (Fishbane et al. 2020; Vernon et al. 2021). Itch‐related QoL data measured with the Skindex‐10 scale have previously been reported and are, therefore, not described in this analysis (Ständer et al. 2024).

### 3.4 Schedule of Assessment for PRO Questionnaires

PROs were assessed during the run‐in period before the first treatment to establish baseline itch severity, and throughout the 12‐week treatment period to measure the antipruritic effects of difelikefalin. The current analysis focuses on the assessments at baseline (day 1) and week 12. Patient‐reported questionnaires were completed preferably within 1 h of starting an HD session and before the completion of each session, and before administration of the study drug. On days when multiple questionnaires were administered, they were completed in the following order: WI‐NRS, SQ‐NRS and then 5‐D itch.

The WI‐NRS and SQ‐NRS questionnaires were completed at each HD session during the run‐in period and before administration of the first dose of study drug on day 1 of the treatment period. They were also completed at each HD session during week 12 and at the end of treatment visit, which occurred at the first HD session after the last dose of study drug had been administered (i.e., the first HD session of week 13). The 5‐D itch scale was completed before the first dose was administered on day 1 and at the end of treatment visit (Supporting Information S1: Figure S1).

### 3.5 Statistical Analysis

Summary statistics are reported as *n*, mean (standard deviation [SD]) for baseline, week 12 and change from baseline to week 12 scores (including 95% confidence intervals [CI] for change from baseline). The proportions of participants with specific improvements in week 12 scores, as well as corresponding 95% CI, were derived from logistic regression models including a term for baseline itch severity. Calculations were based only on individuals with complete data at these timepoints; those with ‘missing data’ were not included in the counts and percentages, except for when reporting WI‐NRS ‘complete responder’ data. In that instance, a conservative approach was taken, and individuals with missing data were counted among ‘non‐complete responders’ to avoid skewing the data.

### 3.6 Statement of Ethical Approval

Study 3105 was approved by the institutions' ethical approval review boards with the Western Institutional Review Board‐Copernicus Group Institutional Review Board (IRB) serving as the central IRB (contract number 20190622). This study was conducted in accordance with the principles of the Declaration of Helsinki. All participants gave written informed consent before study enrolment. No additional consent was required for this exploratory subgroup analysis.

## 4 Results

### 4.1 Individuals With Moderate CKD‐aP at Baseline (WI‐NRS < 7)

#### 4.1.1 Study Disposition, Baseline Demographics and Symptom Burden at Baseline

**Figure 1:**
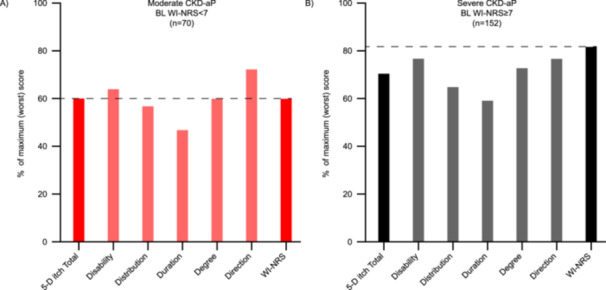
Itch‐related burden at baseline measured by the 5‐D itch scale and WI‐NRS among participants with (A) moderate or (B) severe CKD‐aP at baseline. BL, baseline; CKD‐aP, chronic kidney disease‐associated pruritus; WI‐NRS, worst itching intensity numerical rating scale. Itch‐related burden was assessed at baseline among participants in the (A) ‘moderate’ and (B) ‘severe’ CKD‐aP subgroups using the 5‐D itch scale and the 24‐h WI‐NRS scale. Mean scores for each scale (total scores, and individual domains for 5‐D itch) were calculated and then presented as a percentage of the maximum (worst) possible score for each scale. For 5‐D itch total score and direction domain, data are presented for 69 participants in the moderate subgroup and 149 participants in the severe subgroup; for disability, distribution, duration and degree domains, data are presented for 69 participants in the moderate subgroup and 150 participants in the severe subgroup. WI‐NRS data are presented for all participants in each subgroup.

Of 222 participants in the overall study population who received at least one dose of difelikefalin, 70 (31.5%) were classed as having moderate CKD‐aP at baseline (Table 2); 88.6% of these individuals completed treatment. Mean (SD) age was 57.1 (13.3) years, and 64.3% were males (Table 2). Mean (SD) baseline itch‐related scores were 6.0 (0.5) for the WI‐NRS, 15.2 (3.1) for the 5‐D itch total score, 4.9 (1.9) for the SQ‐NRS and 3.0 (1.2) for the 5D SDQ, indicating itch‐related burden and deficits in itch‐related QoL (Table 2 and Figure 1A). The mean (SD) duration of pruritus was 3.8 (3.3) years; 67.1% of participants in this subgroup were not using anti‐itch medication at baseline (Table 2).

**Table 2:** Study disposition, baseline demographics and clinical characteristics according to baseline itch severity.

	Moderate CKD‐aP BL WI‐NRS < 7 (*n* = 70)	Severe CKD‐aP BL WI‐NRS ≥ 7 (*n* = 152)	Overall study population (*n* = 222)
Study disposition			
Enroled, *n*	70	152	286
Safety population^a^, *n*	70	152	222
Completed treatment, *n* (%)	62 (88.6)	135 (88.8)	197 (88.7)
Discontinued treatment, *n* (%)	8 (11.4)	17 (11.2)	25 (11.3)
Adverse event	5 (7.1)	8 (5.3)	13 (5.9)
Lack of therapeutic efficacy	0	1 (0.7)	1 (0.5)
Lost to follow‐up	0	1 (0.7)	1 (0.5)
Subject withdrew consent	3 (4.3)	4 (2.6)	7 (3.2)
Administrative	0	1 (0.7)	1 (0.5)
Other	0	2 (1.3)	2 (0.9)
Baseline demographics			
Age, years			
Mean (SD)	57.1 (13.3)	58.6 (12.6)	58.1 (12.8)
Sex, *n* (%)			
Female	25 (35.7)	76 (50.0)	101 (45.5)
Male	45 (64.3)	76 (50.0)	121 (54.5)
Ethnicity, *n* (%)			
Hispanic or Latino	12 (17.1)	36 (23.7)	48 (21.6)
Not Hispanic or Latino	58 (82.9)	115 (75.7)	173 (77.9)
Not reported	0 (0)	1 (0.7)	1 (0.5)
Clinical characteristics, mean (SD)			
Baseline WI‐NRS score^b^	6.0 (0.5)	8.3 (0.9)	7.6 (1.3)
Baseline 5‐D itch score^c^	15.2 (3.1)	17.9 (3.3)	17.1 (3.5)
Baseline SQ‐NRS score^b^	4.9 (1.9)	7.4 (1.8)	6.6 (2.2)
Baseline 5D SDQ score^d^	3.0 (1.2)	3.8 (1.0)	3.5 (1.15)
Years on chronic HD	5.3 (4.5)	5.5 (4.4)	5.4 (4.4)
Duration of CKD‐aP, years	3.8 (3.3)	4.0 (3.3)	3.9 (3.3)
Anti‐itch medication use, *n* (%)			
Yes	23 (32.9)	47 (30.9)	70 (31.5)
No	47 (67.1)	105 (69.1)	152 (68.5)

Abbreviations: 5D SDQ, 5‐D itch sleep disability question; BL, baseline; CKD‐aP, chronic kidney disease‐associated pruritus; HD, haemodialysis; SD, standard deviation; SQ‐NRS, sleep quality numerical rating scale; WI‐NRS, worst itching intensity numerical rating scale.

^a^ The term ‘safety population’ was assigned to all participants who received at least one dose of the study medication.

^b^ WI‐NRS and SQ‐NRS data were based on weekly mean scores; data are shown for all participants in each subgroup.

^c^ For baseline 5‐D itch scores, data are reported for 69 participants in the moderate subgroup, 149 participants in the severe subgroup and 218 participants for the overall study population.

^d^ Baseline 5D SDQ scores are reported for 69 participants in the moderate subgroup, 150 participants in the severe subgroup and 219 participants for the overall study population.

#### 4.1.2 Safety

Just over 60% of participants with moderate CKD‐aP at baseline experienced a TEAE during the treatment period (64.3%; Table 3). However, the number of participants who experienced a TEAE leading to the discontinuation of the study drug was relatively low (7.1%; Table 3). In this subgroup, 7.1% experienced an AESI during the study period (Supporting Information S2: Table S2), and one person died during the study, although this death was not considered to be treatment‐related.

**Table 3:** Summary of TEAEs during treatment period according to baseline itch severity.

	Moderate CKD‐aP BL WI‐NRS < 7 (*n* = 70)	Severe CKD‐aP BL WI‐NRS ≥ 7 (*n* = 152)	Overall study population (*n* = 222)
Number of TEAEs	107	307	414
Number of participants with any TEAE, *n* (%)	45 (64.3%)	98 (64.5%)	143 (64.4%)
Number of nonfatal serious TEAEs	16	72	88
Number of participants with any nonfatal serious TEAEs, *n* (%)	9 (12.9%)	35 (23.0%)	44 (19.8%)
Number of participants with any TEAE leading to death, *n* (%)	1 (1.4%)	2 (1.3%)	3 (1.4%)
Number of TEAEs leading to study drug discontinuation	11	10	21
Number of participants with any TEAE leading to study drug discontinuation, *n* (%)	5 (7.1%)	9 (5.9%)	14 (6.3%)
Number of TEAEs leading to study drug interruption	2	48	50
Number of participants with any TEAE leading to study drug interruption, *n* (%)	2 (2.9%)	21 (13.8%)	23 (10.4%)
Number of severe TEAEs	9	23	32
Number of participants with any severe TEAE, *n* (%)	5 (7.1%)	14 (9.2%)	19 (8.6%)
Number of related TEAE reported	8	10	18
Number of participants with any related TEAE, *n* (%)	7 (10.0%)	9 (5.9%)	16 (7.2%)
Number of related serious TEAE reported	0	0	0
Number of participants with any related serious TEAE, *n* (%)	0	0	0
Number of TEAEs of special interest	7	22	29
Number of participants with any TEAEs of special interest, *n* (%)	5 (7.1%)	18 (11.8%)	23 (10.4%)

Abbreviations: BL, baseline; CKD‐aP, chronic kidney disease‐associated pruritus; TEAE, treatment emergent adverse event; WI‐NRS, Worst Itching Intensity Numerical Rating Scale.

#### 4.1.3 Effectiveness

**Figure 2:**
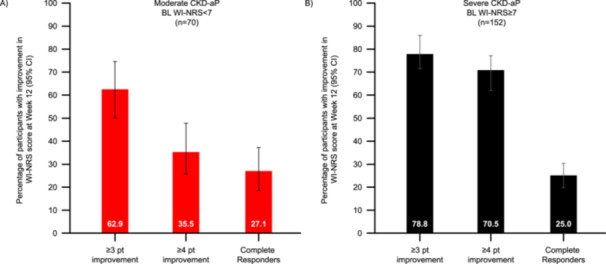
Clinically relevant reductions in WI‐NRS score by week 12 among participants with (A) moderate or (B) severe CKD‐aP at baseline. BL, baseline; CI, confidence interval; CKD‐aP, chronic kidney disease‐associated pruritus; WI‐NRS, worst itching intensity numerical rating scale. Itch intensity was assessed using the 24‐h WI‐NRS scale. The proportion of participants achieving clinically relevant ≥ 3‐point improvements, ≥ 4‐point improvements, or a complete response (≥ 75% of weekly mean WI‐NRS scores equal to 0 or 1) in WI‐NRS scores at week 12 were reported for the (A) ‘moderate’ and (B) ‘severe’ CKD‐aP subgroups. Data are shown as 95% CI derived from a logistic regression model including a term for baseline itch severity. WI‐NRS ≥ 3 and ≥ 4 pt responder data are presented for 62 participants in the moderate subgroup and 132 participants in the severe subgroup; for complete responder data, those with missing data were considered non‐complete responders.

**Figure 3:**
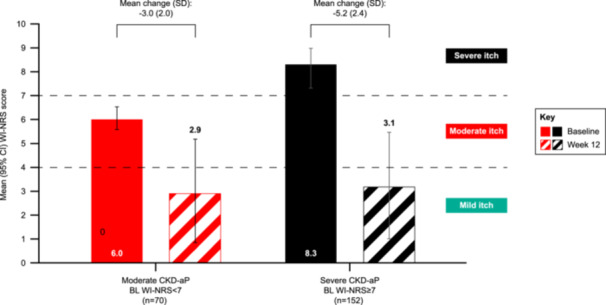
WI‐NRS score at baseline and week 12, and change from baseline to week 12. BL, baseline; CKD‐aP, chronic kidney disease‐associated pruritus; SD, standard deviation; WI‐NRS, worst itching intensity numerical rating scale. Itch intensity was assessed at baseline and week 12 for both severity subgroups using the 24‐h WI‐NRS scale and the mean change from baseline to week 12 in WI‐NRS score was calculated. Week 12 and change from baseline data are presented for 62 participants in the moderate subgroup, and 132 participants in the severe subgroup; baseline data are presented for all participants in each subgroup. Figure modified from Weiner et al. (2022, p. 682), https://doi.org/10.1681/asn.20223311s1682b. Reused with permission obtained from the American Society of Nephrology.

Approximately two‐thirds of participants (62.9%) in the moderate CKD‐aP subgroup achieved a ≥ 3‐point clinically relevant improvement in itch intensity by week 12, as assessed by reductions in mean WI‐NRS scores (Figure 2A). With regard to a ≥ 4‐point clinically relevant improvement in itch intensity, 35.5% reached this improvement threshold by week 12, and 27.1% demonstrated a ‘complete response’ according to WI‐NRS score (Figure 2A). The mean (SD) change from baseline to week 12 in WI‐NRS score was −3.0 (2.0) among individuals in this subgroup (95% CI: −3.6 to −2.5), and the mean (SD) score at week 12 was 2.9 (2.2), indicating residual ‘mild‐itch’ following 12 weeks of treatment with difelikefalin (Figure 3).

**Figure 4:**
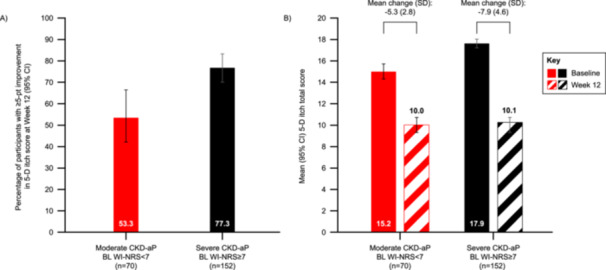
Clinically relevant improvements in 5‐D itch total score at week 12 (A); 5‐D itch total score at baseline and week 12 (B). BL, baseline; CI, confidence interval; CKD‐aP, chronic kidney disease‐associated pruritus; SD, standard deviation; WI‐NRS, worst itching intensity numerical rating scale. (A) The proportion of participants in either severity subgroup achieving clinically relevant ≥ 5‐pt improvements in itch‐related quality of life by week 12 was assessed using the 5‐D itch scale, and data presented as 95% CI derived from a logistic regression model including a term for baseline itch severity. ≥ 5‐pt responder data are shown for 60 participants in the moderate subgroup and 132 participants in the severe subgroup. (B) Mean 5‐D itch total scores were assessed at baseline and week 12 for both severity subgroups and the mean change from baseline to week 12 was calculated. Baseline data are presented for 69 participants in the moderate subgroup and 149 in the severe subgroup; Week 12 data, for 61 participants in the moderate subgroup and 134 in the severe subgroup; and change from baseline data, for 60 participants in the moderate subgroup and 132 participants in the severe subgroup.

More than half of all participants with moderate CKD‐aP at baseline (53.3%) achieved a ≥ 5‐point improvement on the 5‐D itch scale total score, indicating clinically relevant improvements in itch‐related QoL (Figure 4A). Furthermore, the mean (SD) change from baseline to week 12 in 5‐D itch total score was −5.3 (2.8) among this subgroup (95% CI: −6.0 to −4.5), and the mean (SD) score at week 12 was 10.0 (3.4) (Figure 4B).

**Figure 5:**
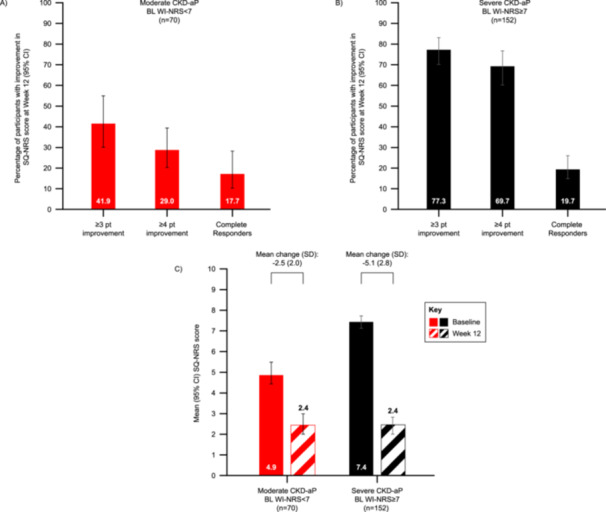
Clinically relevant improvements in SQ‐NRS score at week 12 among participants with (A) moderate or (B) severe CKD‐aP at baseline; SQ‐NRS scores at baseline and week 12 (C). BL, baseline; CI, confidence interval; CKD‐aP, chronic kidney disease‐associated pruritus; SD, standard deviation; SQ‐NRS, sleep quality numerical rating scale; WI‐NRS, worst itching intensity numerical rating scale. Itch‐related sleep quality was assessed at baseline and following 12 weeks of treatment with difelikefalin using the SQ‐NRS. The proportion of participants achieving clinically relevant ≥ 3 or ≥ 4‐point improvements, or a complete response (weekly mean SQ‐NRS scores equal to 0 at week 12) in SQ‐NRS scores at week 12 were reported for the (A) ‘moderate’ and (B) ‘severe’ subgroups. Data are presented as 95% CI derived from a logistic regression model including a term for baseline itch severity. Mean SQ‐NRS scores for both severity subgroups were reported at baseline and Week 12, and the mean change from baseline to Week 12 was calculated (C). Data shown here for 62 participants in the moderate subgroup and 132 participants in the severe subgroup, with the exception of baseline mean score data, which is presented for all participants in each subgroup.

Improvements in itch‐related sleep quality were also noted among participants with moderate CKD‐aP at baseline, with 41.9% and 29.0% reaching clinically relevant ≥ 3 and ≥ 4‐point improvements in SQ‐NRS scores, respectively. Additionally, 17.7% demonstrated a ‘complete response’ with regard to improved sleep quality (Figure 5A). The mean (SD) change from baseline in SQ‐NRS score was −2.5 (2.0) among these individuals (95% CI: −3.0 to −2.0), and the mean (SD) SQ‐NRS score at week 12 was 2.4 (2.2) (Figure 5C). Furthermore, 59.1% reported a ≥ 1‐point improvement in 5D SDQ score after 12 weeks on difelikefalin (data not shown). By week 12, the mean (SD) change from baseline in 5D SDQ score was −1.0 (1.4) for participants in this subgroup (95% CI: −1.4 to −0.7), and the week 12 mean (SD) score was 2.0 (1.1), indicating ‘occasional delays falling asleep’ (data not shown).

### 4.2 Individuals With Severe CKD‐aP at Baseline (WI‐NRS ≥ 7)

#### 4.2.1 Study Disposition, Baseline Demographics and Symptom Burden at Baseline

One hundred and fifty‐two participants (68.5%) in Study 3105 had severe CKD‐aP at baseline; 88.8% completed treatment with difelikefalin (Table 2). The mean (SD) age in this subgroup was 58.6 (12.6) years, and 50% were males (Table 2). Substantial itch‐related burden at baseline was evidenced by mean (SD) itch‐related scores of 8.3 (0.9) for the WI‐NRS, 17.9 (3.3) for the 5‐D itch scale total score, 7.4 (1.8) for the SQ‐NRS and 3.8 (1.0) for the 5D SDQ (Table 2 and Figure 1B). The mean (SD) duration of pruritus was 4.0 (3.3) years; 69.9% of individuals were not using anti‐itch medication at baseline (Table 2).

#### 4.2.2 Safety

Among those with severe CKD‐aP at baseline, 64.5% experienced a TEAE during the treatment period, but only 5.9% experienced a TEAE, which led to the discontinuation of the study drug (Table 3 *)*. AESI were experienced by 11.8% of participants in this subgroup (Supporting Information S2: Table S2), and two people (1.3%) died during the study, although these deaths were not considered to be treatment‐related (Table 3).

#### 4.2.3 Effectiveness

Almost 80% of individuals with severe CKD‐aP at baseline (78.8%) achieved a ≥ 3‐point clinically relevant improvement in itch intensity by week 12, with 70.5% achieving a ≥ 4‐point clinically relevant improvement, as assessed by reductions in mean WI‐NRS scores. A quarter of participants (25.0%) had a ‘complete response’ by week 12 according to WI‐NRS score (Figure 2B). The mean (SD) change from baseline to week 12 in WI‐NRS score was −5.2 (2.4) among this subgroup (95% CI: −5.6 to −4.8). After 12 weeks of treatment with difelikefalin, mean (SD) WI‐NRS score was 3.1 (2.3), indicative of residual ‘mild‐itch’ (Figure 3).

Similarly, over three‐quarters of individuals in the severe subgroup (77.3%) reached a ≥ 5‐point improvement on the 5‐D itch scale total score, indicating clinically relevant improvements in itch‐related QoL (Figure 4A). Moreover, the mean (SD) change from baseline to week 12 in 5‐D itch total score was −7.9 (4.6) among this subgroup (95% CI: −8.7 to −7.1), and the mean (SD) 5‐D itch total score at week 12 was 10.1 (3.5) (Figure 4B).

With regard to sleep quality, 77.3% of those with severe CKD‐aP achieved a ≥ 3‐point improvement in SQ‐NRS scores by week 12, with 69.7% reaching a ≥ 4‐point improvement in this time frame (Figure 5B). One‐fifth of participants (19.8%) demonstrated a ‘complete response’ according to SQ‐NRS scores by week 12 (Figure 5B). The mean (SD) change from baseline in SQ‐NRS score was −5.1 (2.8) among these individuals (95% CI: −5.5 to −4.6), and the mean (SD) SQ‐NRS score at week 12 was 2.4 (2.2) (Figure 5C). Additionally, 82.7% reported a ≥ 1‐point improvement in 5D SDQ score after 12 weeks of difelikefalin treatment (data not shown). By week 12, the mean (SD) change from baseline in 5D SDQ score was −1.8 (1.3) among this subgroup (95% CI: −2.0 to −1.6), and the week 12 mean (SD) score was 2.0 (1.1), indicative of ‘occasional delays falling asleep’ (data not shown).

## 5 Discussion

This exploratory subgroup analysis from Study 3105 demonstrates that difelikefalin, a first‐in‐class drug approved for the treatment of moderate‐to‐severe CKD‐aP among adults receiving maintenance HD (US Food and Drug Administration 2021), appears to be a well‐tolerated and effective treatment, irrespective of baseline itch severity (Fishbane et al. 2020; Topf et al. 2022; Weiner et al. 2022; 2024). Specifically, difelikefalin reduced itch intensity and improved itch‐related QoL and sleep quality among adults on HD with either moderate CKD‐aP (WI‐NRS < 7) or severe CKD‐aP (WI‐NRS ≥ 7) at baseline.

Furthermore, safety and effectiveness outcomes were similar between participants in both subgroups, regardless of their baseline itch severity, supporting the use of this treatment in a broad range of individuals on HD who have CKD‐aP. These findings are consistent with data from two previously published phase 3 placebo‐controlled trials (KALM‐1 and KALM‐2) and the primary analysis from Study 3105, which demonstrated the tolerable safety profile and effectiveness of difelikefalin in significantly improving itch‐related outcomes among adults on HD with moderate‐to‐severe CKD‐aP (Fishbane et al. 2020; Topf et al. 2022; Weiner et al. 2022).

In the present analysis, baseline demographics were closely aligned between participants in either CKD‐aP severity subgroup, with the exception that there were more males than females in the moderate subgroup. Given the lack of consistent evidence to support the existence of sex differences in the pathophysiology of CKD‐aP, it was concluded that the higher proportion of males in the moderate versus severe subgroup should not influence our findings (Martin et al. 2020). Therefore, any observed differences in treatment outcomes should be attributable to differences in baseline itch severity.

As anticipated, individuals with severe CKD‐aP reported worse baseline itch‐related QoL scores than those with moderate CKD‐aP. However, both subgroups demonstrated considerable itch‐related burden at baseline, as well as detriments to itch‐related QoL and sleep quality and the duration of CKD‐aP was similar between subgroups. Despite this obvious burden, anti‐itch medication use was similarly low across both subgroups, with approximately 70% of participants in either subgroup reportedly not taking any anti‐itch medication. Thus, there is a clear need to prescribe effective treatment for individuals with moderate CKD‐aP, as well as those with more severe itch.

With regard to safety, the occurrence of nonfatal serious TEAEs, TEAEs leading to discontinuation of the study drug and AESIs was considered to be relatively low across both subgroups, irrespective of baseline itch severity. Importantly, death occurred in only 1% of participants in either subgroup during the treatment period and these deaths were not considered to be treatment‐related. Therefore, our findings indicate that there were no new safety concerns associated with difelikefalin treatment according to baseline itch severity. Taken together, these data demonstrate that difelikefalin was well‐tolerated in adults receiving maintenance HD, with either moderate or severe CKD‐aP, in line with previous safety findings (Fishbane et al. 2022; Weiner et al. 2022).

Clinically meaningful reductions in itch intensity (WI‐NRS) and improvements in itch‐related QoL (5‐D itch) and sleep quality (SQ‐NRS, 5D SDQ) were evidenced in individuals from both baseline severity subgroups after 12 weeks of treatment with difelikefalin. Notably, by week 12, both subgroups achieved almost analogous mean WI‐NRS, 5‐D itch total, SQ‐NRS, and 5D SDQ scores, indicative of comparably low itch intensity and improved itch‐related QoL and sleep quality following treatment. Although the proportion of participants achieving specific thresholds of improvement was greater among individuals in the severe subgroup versus those in the moderate subgroup, as were the mean change from baseline data, it is likely that this was due to the fact that baseline itch and itch‐related QoL scores were higher (worse) among the severe group to begin with. This may have meant that lower baseline scores among participants in the moderate subgroup created a ceiling effect, limiting the potential magnitude for improvement.

Nonetheless, the fact that both subgroups were left with, on average, residual ‘mild itch’ and only minimal itch‐related sleep disturbances and itch‐related QoL burden by week 12, irrespective of their baseline itch severity, suggests comparable treatment outcomes for those with moderate or severe CKD‐aP. This observation is further supported by our ‘complete responder’ data, which shows that the proportion of participants achieving a complete response in terms of itch relief or improvements in sleep quality by week 12 was also similar between both subgroups, at approximately 25%–27% for WI‐NRS and 18%–20% for SQ‐NRS, suggesting comparable benefits for individuals with moderate or severe CKD‐aP.

One possible limitation of the overall study design for Study 3105 is the lack of placebo control. However, as previously described (Weiner et al. 2022), the open‐label, single‐arm design of Study 3105 more closely reflects a real‐world treatment scenario, potentially making findings more relatable to a clinical setting. Furthermore, overall observations from Study 3105 closely reflected previous findings from placebo‐controlled trials, demonstrating consistent antipruritic effects of difelikefalin among this patient group (Fishbane et al. 2020; Topf et al. 2022). It is also important to note that as with any secondary analysis, this study was exploratory in nature; therefore, findings must be interpreted appropriately. Nonetheless, this analysis provides valuable and novel insights into the potential impact of differences in baseline itch severity on treatment outcomes for difelikefalin, which complement previous safety and effectiveness data (Fishbane et al. 2020; Topf et al. 2022; Weiner et al. 2022, 2024).

### 5.1 Implications for Clinical Practice

There is a clear clinical need for effective anti‐itch treatment to improve itch‐related QoL among those with moderate, as well as severe, CKD‐aP, as demonstrated by our observation of substantial itch‐related burden and deficits in itch‐related QoL among both groups of participants in this study. Our findings confirm the safety and effectiveness of difelikefalin in adults on maintenance HD with either moderate or severe CKD‐aP at baseline. These findings should be considered in clinical practice to support evidence‐based decision‐making for the treatment of CKD‐aP among individuals with varying itch severity, ensuring that those with moderate as well as severe CKD‐aP are prioritised for treatment.

Healthcare staff who are considering prescribing difelikefalin should confirm that the intended patient is receiving in‐centre HD and has moderate‐to‐severe CKD‐aP, determined using a single WI‐NRS measurement. A single WI‐NRS measurement should then be taken after 12 weeks of treatment with difelikefalin to confirm clinical efficacy. The reliability and validity of a single measure of WI‐NRS to determine itch intensity, compared to the mean of several WI‐NRS measurements, has previously been demonstrated (McCafferty et al. 2024). Moreover, in clinical practice, this single‐measure approach offers improved efficiency.

Evidence to support the efficacy and tolerability of difelikefalin has been established for up to 1 year (Fishbane et al. 2020). In line with the findings in this article, the most common adverse events in those receiving difelikefalin for up to a year were diarrhoea, dizziness and vomiting (Fishbane et al. 2020). These adverse events were generally mild‐to‐moderate in severity and resolved without evident clinical consequence (Fishbane et al. 2020).

## 6 Conclusions

Difelikefalin was well‐tolerated among adults currently receiving maintenance HD, who have either moderate or severe CKD‐aP, with no new safety concerns associated with treatment on the basis of baseline itch severity. Treatment with difelikefalin resulted in clinically relevant improvements in itch reduction, itch‐related QoL and itch‐related sleep quality among all participants by week 12, regardless of the severity of their itch at baseline. In both the moderate and severe subgroups, residual itch intensity, itch‐related QoL burden and itch‐related sleep detriments were comparably low (improved) after 12 weeks of treatment with difelikefalin. Moreover, the proportion of participants achieving a ‘complete response’ in terms of itch relief and improved itch‐related sleep quality was similar between the moderate and severe subgroups. Taken together, these data indicate similar treatment outcomes and effectiveness of difelikefalin among participants in either severity subgroup, irrespective of baseline itch severity. Overall, our findings support the use of difelikefalin treatment in a broad range of individuals on maintenance HD with CKD‐aP, regardless of whether their baseline itch is moderate or severe.

## Author Contributions

The principles of Good Publication Practice were followed in the development of this manuscript. All authors had access to the study data throughout and fulfilled the ICMJE criteria for authorship by contributing to the preparation of this manuscript and by providing approval of the final version. K.M. was responsible for data analysis and interpretation. T.S. and W.W. were responsible for the concept and design of the study, data acquisition and data analysis and interpretation. P.M. was responsible for data analysis and interpretation. M.L. was responsible for data analysis and interpretation. D.W. was responsible for data acquisition and data analysis and interpretation.

## Conflicts of Interest

K.M. and P.M. have received consultation and speaker fees from CSL Vifor. T.S. and M.L. are employees of CSL Vifor. W.W. is an employee of Cara Therapeutics. D.W. declare no conflicts of interest.

## Supporting information

J of Renal Care 3105 Baseline Severity ms Supp Fig 1.pdf.

Revised supplementary Study 3105 Baseline severity Final March 2025.

## Data Availability

The data underlying this article will be shared on reasonable request by the corresponding author.

## References

[jorc70017-bib-0001] Elman, S. , L. S. Hynan , V. Gabriel , and M. J. Mayo . 2010. “The 5‐D Itch Scale: A New Measure of Pruritus.” British Journal of Dermatology 162, no. 3: 587–593. 10.1111/j.1365-2133.2009.09586.x. 19995367 PMC2875190

[jorc70017-bib-0002] European Medicines Agency . 2022. *Kapruvia (Difelikefalin): An Overview of Kapruvia and Why It Is Authorised in the EU*. Accessed October, 2024. https://www.ema.europa.eu/en/documents/overview/kapruvia-epar-medicine-overview_en.pdf.

[jorc70017-bib-0003] Fishbane, S. , A. Jamal , C. Munera , W. Wen , and F. Menzaghi . 2020. “A Phase 3 Trial of Difelikefalin in Hemodialysis Patients With Pruritus.” New England Journal of Medicine 382, no. 3: 222–232. 10.1056/NEJMoa1912770. 31702883

[jorc70017-bib-0004] Fishbane, S. , W. Wen , C. Munera , et al. 2022. “Safety and Tolerability of Difelikefalin for the Treatment of Moderate to Severe Pruritus in Hemodialysis Patients: Pooled Analysis From the Phase 3 Clinical Trial Program.” Kidney Medicine 4, no. 8: 100513. 10.1016/j.xkme.2022.100513. 36039153 PMC9418597

[jorc70017-bib-0005] Kim, D. , and C. Pollock . 2021. “Epidemiology and Burden of Chronic Kidney Disease‐Associated Pruritus.” Clinical Kidney Journal 14, no. Suppl 3: i1–i7. 10.1093/ckj/sfab142. PMC8702817 34987777

[jorc70017-bib-0006] Kumagai, H. , T. Ebata , K. Takamori , T. Muramatsu , H. Nakamoto , and H. Suzuki . 2010. “Effect of a Novel Kappa‐Receptor Agonist, Nalfurafine Hydrochloride, on Severe Itch in 337 Haemodialysis Patients: A Phase III, Randomized, Double‐Blind, Placebo‐Controlled Study.” Nephrology Dialysis Transplantation 25, no. 4: 1251–1257. 10.1093/ndt/gfp588. 19926718

[jorc70017-bib-0007] Martin, C. E. , S. Clotet‐Freixas , J. F. Farragher , and G. L. Hundemer . 2020. “Have We Just Scratched the Surface? A Narrative Review of Uremic Pruritus in 2020.” Canadian Journal of Kidney Health and Disease 7: 2054358120954024. 10.1177/2054358120954024. 33117546 PMC7573751

[jorc70017-bib-0008] Mathur, V. S. , J. Lindberg , M. Germain , et al. 2010. “A Longitudinal Study of Uremic Pruritus in Hemodialysis Patients.” Clinical Journal of the American Society of Nephrology 5, no. 8: 1410–1419. 10.2215/CJN.00100110. 20558560 PMC2924419

[jorc70017-bib-0009] McCafferty, K. , T. Schaufler , I. Morin , W. Wen , and L. Chan . 2024. “Validity of a Single Worst Itch Numeric Rating Scale (WI‐NRS) Measurement to Identify Patients With Moderate or Severe CKD‐Associated Pruritus. Congress Abstract. ASN TH‐P0297.” Journal of the American Society of Nephrology 35–S. 10.1681/ASN.2024k64q3cs2.

[jorc70017-bib-0010] Rayner, H. C. , M. Larkina , M. Wang , et al. 2017. “International Comparisons of Prevalence, Awareness, and Treatment of Pruritus in People on Hemodialysis.” Clinical Journal of the American Society of Nephrology 12, no. 12: 2000–2007. 10.2215/CJN.03280317. 28923831 PMC5718267

[jorc70017-bib-0011] Rehman, I. U. , K. G. Chan , S. Munib , L. H. Lee , and T. M. Khan . 2019. “The Association Between CKD‐Associated Pruritus and Quality of Life in Patients Undergoing Hemodialysis in Pakistan: A Strobe Complaint Cross‐Sectional Study.” Medicine 98, no. 36: e16812. 10.1097/MD.0000000000016812. 31490367 PMC6739024

[jorc70017-bib-0012] Ständer, S. , M. Augustin , A. Reich , et al. 2013. “Pruritus Assessment in Clinical Trials: Consensus Recommendations From the International Forum for the Study of Itch (IFSI) Special Interest Group Scoring Itch in Clinical Trials.” Acta Dermato Venereologica 93, no. 5: 509–514. 10.2340/00015555-1620. 23624777

[jorc70017-bib-0013] Ständer, S. , S. Fishbane , T. Schaufler , et al. 2024. “Chronic Kidney Disease‐Associated Pruritus and Quality of Life With Difelikefalin Treatment: A Post Hoc Analysis of Phase 3 Data Using the Skindex‑10 Questionnaire.” Clinical Kidney Journal 17, no. 10: sfae274. 10.1093/ckj/sfae274. 39421431 PMC11484513

[jorc70017-bib-0014] Sukul, N. , A. Karaboyas , P. A. Csomor , et al. 2021. “Self‐Reported Pruritus and Clinical, Dialysis‐Related, and Patient‐Reported Outcomes in Hemodialysis Patients.” Kidney Medicine 3, no. 1: 42–53.e1. 10.1016/j.xkme.2020.08.011. 33604539 PMC7873756

[jorc70017-bib-0015] Topf, J. , T. Wooldridge , K. McCafferty , et al. 2022. “Efficacy of Difelikefalin for the Treatment of Moderate to Severe Pruritus in Hemodialysis Patients: Pooled Analysis of KALM‐1 and KALM‐2 Phase 3 Studies.” Kidney Medicine 4, no. 8: 100512. 10.1016/j.xkme.2022.100512. 36016762 PMC9396406

[jorc70017-bib-0016] US Food and Drug Administration (FDA) . 2021. KORSUVA (Difelikefalin): Highlights of Prescribing Information. Accessed October, 2024. https://www.accessdata.fda.gov/drugsatfda_docs/label/2021/214916s000lbl.pdf.

[jorc70017-bib-0017] Vernon, M. , S. Ständer , C. Munera , R. H. Spencer , and F. Menzaghi . 2021. “Clinically Meaningful Change in Itch Intensity Scores: An Evaluation In Patients With Chronic Kidney Disease‐Associated Pruritus.” Journal of the American Academy of Dermatology 84, no. 4: 1132–1134. 10.1016/j.jaad.2020.06.991. 32603719

[jorc70017-bib-0018] Weiner, D. E. , T. Schaufler , K. McCafferty , et al. 2024. “Difelikefalin Improves Itch‐Related Sleep Disruption in Patients Undergoing Haemodialysis.” Nephrology Dialysis Transplantation 39, no. 7: 1125–1137. 10.1093/ndt/gfad245. PMC11210984 37968132

[jorc70017-bib-0019] Weiner, D. E. , M. G. Vervloet , S. Walpen , et al. 2022. “Safety and Effectiveness of Difelikefalin in Patients With Moderate‐to‐Severe Pruritus Undergoing Hemodialysis: An Open‐Label, Multicenter Study.” Kidney Medicine 4, no. 10: 100542. 10.1016/j.xkme.2022.100542. 36185706 PMC9516453

