# Supplementary figures and images for: Influence of Baseline Itch Severity on Treatment Outcomes With Difelikefalin in Adults With Moderate‐to‐Severe Pruritus Receiving Maintenance Haemodialysis: An Exploratory Analysis
Source: J Ren Care. 2025 May 6;51(2):e70017. doi: 10.1111/jorc.70017 (PMC12054700; doi:10.1111/jorc.70017)

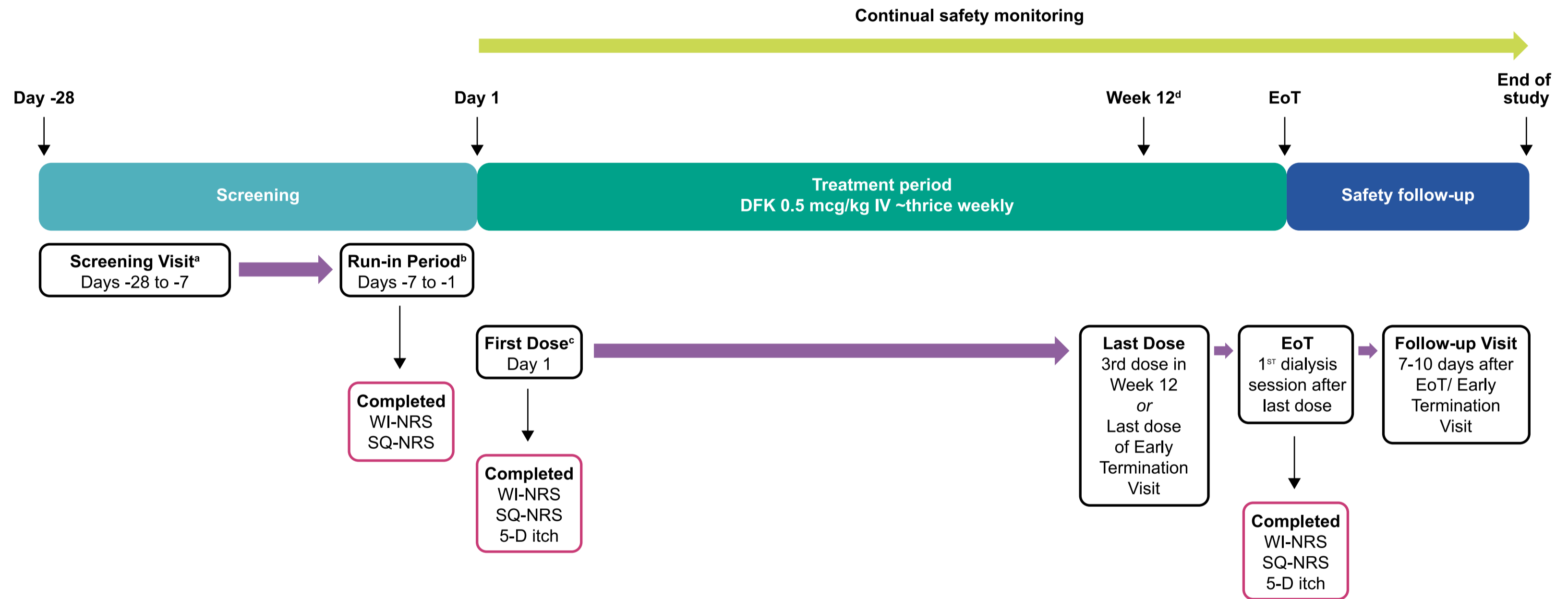

Supplement: Supplementary file 1 — J of Renal Care 3105 Baseline Severity ms Supp Fig 1.pdf. [file JORC-51-0-s002.pdf]
