# Supplementary material for: Influence of Baseline Itch Severity on Treatment Outcomes With Difelikefalin in Adults With Moderate‐to‐Severe Pruritus Receiving Maintenance Haemodialysis: An Exploratory Analysis
Source: J Ren Care. 2025 May 6;51(2):e70017. doi: 10.1111/jorc.70017 (PMC12054700; doi:10.1111/jorc.70017)
Supplement: Supplementary file 2 — Revised supplementary Study 3105 Baseline severity Final March 2025. [file JORC-51-0-s001.docx]

**Supplementary materials**

**Influence of baseline itch severity on treatment outcomes with difelikefalin in adults with moderate-to-severe pruritus receiving maintenance haemodialysis: An exploratory analysis**

**Running title:** Difelikefalin treatment outcomes by itch severity

**Supplementary Table 1. Eligibility criteria for Study 3105**

| **Inclusion criteria** |
| --- |
| 1. Willing and able to provide written informed consent prior to participating in the study 2. Able to communicate clearly with the investigator and staff, able to understand the study procedures, and able and willing to comply with the study schedules and all study requirements 3. Males or females; aged between 18–85 years of age, inclusive 4. Diagnosis of ESRD and been on hemodialysis 3 times per week for at least 3 months prior to the start of screening^a,b,c^ 5. **If assigned female at birth**:    1. Was not pregnant or nursing during any period of the study   **or**   - 1. Was surgically sterile; or had been amenorrheic for at least 1 year and was over the age of 55 years   **or**   - 1. Had a negative serum pregnancy test at screening and agreed to use acceptable contraceptive measures from the time of informed consent until 7 days after the last dose of study drug  1. **If assigned male at birth**, had agreed not to donate sperm after the first dose of study drug until 7 days after the last dose of study drug, and agreed to use a condom with spermicide or abstain from heterosexual intercourse during the study until 7 days after study drug administration^d^ 2. Had a prescription dry body weight of ≥40.0 kg 3. Over the last 3 months prior to screening, had at least 1 of the following:    1. At least 2 single-pool measurements of (dialyzer clearance of urea × dialysis time) / (volume of distribution of urea) of ≥1.2 on different dialysis days    2. At least 2 urea reduction ratio measurements of ≥65% on different dialysis days    3. 1 single-pool measurement of (dialyzer clearance of urea × dialysis time) / (volume of distribution of urea) of ≥1.2 and 1 urea reduction ratio measurement of ≥65% on different dialysis days 4. Prior to treatment:    1. Had completed at least 3 WI-NRS questionnaires from the start of the Run-in Period up to and including the assessment on Day 1    2. Had a mean baseline WI-NRS score of ≥5, defined as the average of all non-missing scores reported from the start of the Run-in Period up to and including the pre-dose assessment on Day 1 |

| **Exclusion criteria** |
| --- |
| 1. Known non-compliance with dialysis treatment that, in the opinion of the investigator, would have impeded completion or validity of the study 2. Was scheduled to receive a kidney transplant during the study 3. Had known history of allergic reaction to opiates, such as hives^e^ 4. Had hypersensitivity to the active substance or any of the excipients in the investigational products 5. Had a concomitant disease or a history of any medical condition that, in the opinion of the investigator, could have posed undue risk to the subject, impeded completion of the study procedures, or compromised the validity of the study measurements, including but not limited to:    1. Known or suspected history of alcohol, narcotic, or other drug abuse, or substance dependence within 12 months prior to screening.    2. Significant systolic or diastolic heart failure (e.g., New York Heart Association Class IV congestive heart failure)    3. Severe mental illness or cognitive impairment (e.g., dementia)    4. Relevant acute or chronic medical or neuropsychiatric condition within 3 months prior to screening (e.g., diagnosis of encephalopathy, coma, delirium) 6. Had received new treatment or changed treatment for itch, including antihistamines and corticosteroids within 14 days prior to screening 7. Had received a new prescription or had a change in prescription for opioids, gabapentin, or pregabalin within 14 days prior to screening 8. Had received another investigational drug within 30 days or 5 half-lives (whichever was longer) prior to the start of dosing or was planning to participate in another interventional clinical study while enrolled in this study 9. In the opinion of the investigator, had pruritus attributed to a cause other than ESRD^f^ or its complications (e.g., subjects with concomitant pruritic dermatological disease or cholestatic liver disease) 10. Had localized itch restricted to the palms of the hands 11. Had pruritus only during the dialysis session (by subject report) 12. Was receiving ongoing ultraviolet B treatment and anticipated receiving such treatment during the study 13. Participated in a previous clinical study with difelikefalin |

ESRD, end-stage renal disease; HD, hemodialysis; WI-NRS, Worst Itching Intensity Numerical Rating Scale.

^a^Participants who required an occasional additional HD treatment to manage fluid overload or electrolyte excesses could be enrolled as long as it was anticipated that no more than 1 such treatment would be required in any given week. ^b^Participants receiving in-home HD could take part as long as they had switched to in-center HD at least 2 weeks prior to screening and planned to remain on in-center HD for the duration of the study. ^c^Participants receiving alternative dialysis modalities, such as nocturnal dialysis, were not eligible. ^d^No restrictions were required for a male who had undergone vasectomy, provided it was performed ≥4 months prior to screening. ^e^Side effects related to the use of opioids, such as constipation or nausea, did not exclude participants from the study. ^f^Participants whose pruritus was attributed to ESRD complications, such as hyperparathyroidism, hyperphosphatemia, anemia, the dialysis procedure, or prescription could be enrolled.

***Key***

**Supplementary Table 2. Summary of AESI by baseline itch severity**

|  | **Moderate CKD-aP BL WI-NRS <7 (n=70)** | **Severe CKD-aP**  **BL WI-NRS ≥7**  **(n=150)** | **Overall study population (n=222)** |
| --- | --- | --- | --- |
| Participants with any AESI, n (%) | 5 (7.1) | 18 (11.8) | 23 (10.4) |
| AESI category^a^, n (%) |  |  |  |
| Dizziness | 2 (2.9) | 5 (3.3) | 7 (3.2) |
| Somnolence | 1 (1.4) | 5 (3.3) | 6 (2.7) |
| Fall | 2 (2.9) | 3 (2.0) | 5 (2.3) |
| Syncope | 0 | 4 (2.6) | 4 (1.8) |
| Mental status changes | 1 (1.4) | 2 (1.3) | 3 (1.4) |
| Palpitations | 0 | 1 (0.7) | 1 (0.5) |
| Seizure | 1 (1.4) | 0 | 1 (0.5) |

AESI, adverse event of special interest; BL, baseline; CKD-aP, chronic kidney disease-associated pruritus; WI-NRS, Worst Itching Intensity Numerical Rating Scale.

^a^Number of participants experiencing an AESI within each category.

**Supplementary Figure 1. Overall study design for Study 3105**


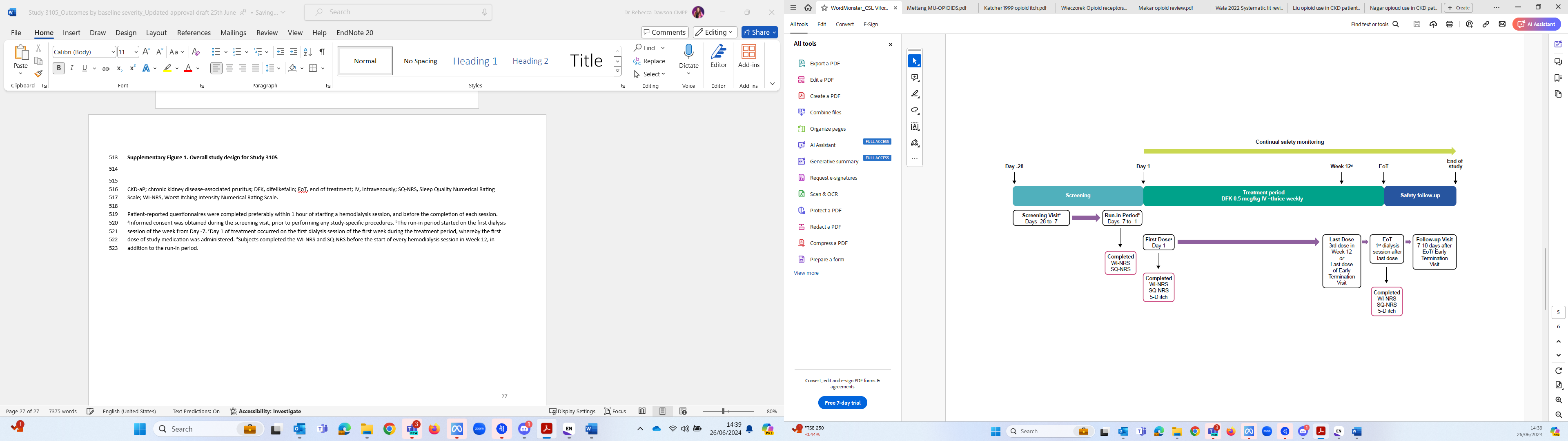


DFK, difelikefalin; EoT, end of treatment; IV, intravenously; SQ-NRS, Sleep Quality Numerical Rating Scale; WI-NRS, Worst Itching Intensity Numerical Rating Scale.

Patient-reported questionnaires were completed preferably within 1 hour of starting a hemodialysis session, and before the completion of each session.
^a^Informed consent was obtained during the screening visit, prior to performing any study-specific procedures. ^b^The run-in period started on the first dialysis session of the week from Day -7. ^c^Day 1 of treatment occurred on the first dialysis session of the first week during the treatment period, whereby the first dose of study medication was administered. ^d^Participants completed the WI-NRS and SQ-NRS before the start of every hemodialysis session in Week 12, in addition to the run-in period.
